# Insights into the molecular mechanism of dehalogenation catalyzed by D-2-haloacid dehalogenase from crystal structures

**DOI:** 10.1038/s41598-017-19050-x

**Published:** 2018-01-23

**Authors:** Yayue Wang, Yanbin Feng, Xupeng Cao, Yinghui Liu, Song Xue

**Affiliations:** 1 Marine Bioengineering Group, Dalian Institute of Chemical Physics, Chinese Academy of Sciences, Dalian, 116023 China; 2 University of Chinese Academy of Sciences, Beijing, 100049 China

## Abstract

D-2-haloacid dehalogenases (D-DEXs) catalyse the hydrolytic dehalogenation of D-2-haloacids, releasing halide ions and producing the corresponding 2-hydroxyacids. A structure-guided elucidation of the catalytic mechanism of this dehalogenation reaction has not been reported yet. Here, we report the catalytic mechanism of a D-DEX, HadD AJ1 from *Pseudomonas putida* AJ1/23, which was elucidated by X-ray crystallographic analysis and the H_2_^18^O incorporation experiment. HadD AJ1 is an α-helical hydrolase that forms a homotetramer with its monomer including two structurally axisymmetric repeats. The product-bound complex structure was trapped with L-lactic acid in the active site, which is framed by the structurally related helices between two repeats. Site-directed mutagenesis confirmed the importance of the residues lining the binding pocket in stabilizing the enzyme-substrate complex. Asp205 acts as a key catalytic residue and is responsible for activating a water molecule along with Asn131. Then, the hydroxyl group of the water molecule directly attacks the C2 atom of the substrate to release the halogen ion instead of forming an enzyme-substrate ester intermediate as observed in L-2-haloacid dehalogenases. The newly revealed structural and mechanistic information on D-DEX may inspire structure-based mutagenesis to engineer highly efficient haloacid dehalogenases.

## Introduction

Dehalogenases are capable of degrading a wide range of halogenated compounds by cleaving the C-X bond. Such enzymes are fascinating for their valuable applications in green chemistry, biocatalysis and bioremediation^1–7^. Dehalogenases currently reported are involved in the cleavage of P-X and C-X bonds in the Brenda database. More than 90% of dehalogenases cleave C-X bonds. Such C-X bonds mainly exist in the halocarbons, halohydrins, and haloacids and their derivatives^8–10^. 2-Haloacid dehalogenases (2-HADs) catalyse the hydrolytic dehalogenation of 2-haloacids, releasing halogen ions and producing corresponding 2-hydroxyacids. 2-HADs are phylogenetically classified into two groups, I and II^11^. Group II enzymes include L-2-haloacid dehalogenases (L-DEXs) which specifically act on L-2-haloacids. D-2-haloacid dehalogenases (D-DEXs) and DL-2-haloacid dehalogenases (DL-DEXs) belong to Group I dehalogenases because of their high similarity in amino acid sequence. D-DEXs specifically act on D-2-haloacids, whereas DL-DEXs act on both D- and L-2-haloacids.

**Figure 1:**
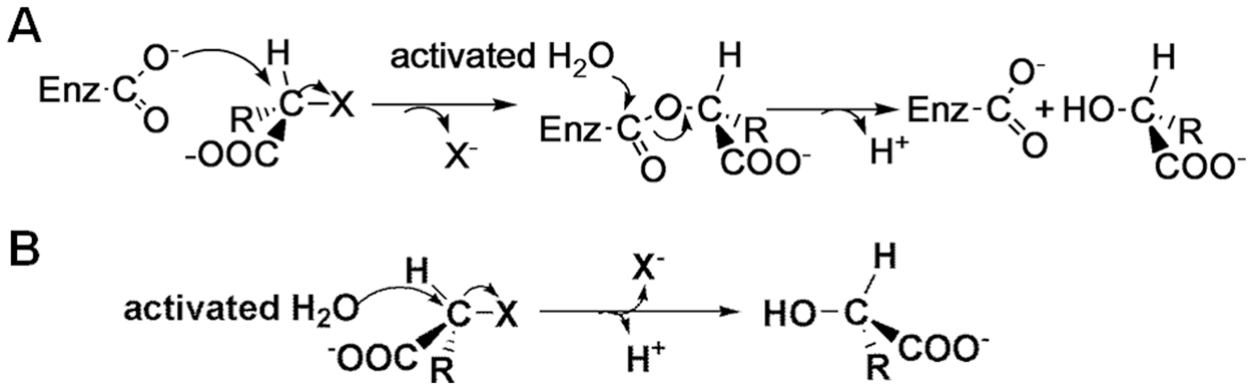
Reaction mechanism of L-DEX and DL-DEX. (**A**) L-DEX: nucleophilic attack by acidic residue followed by formation of an esterified intermediate. (**B**) DL-DEX: a water molecule activated by the enzyme directly attacks the C2 atom of the substrate without forming an E-S ester intermediate.

L-DEX has been intensively studied because of its abundance in nature. It belongs to a call of α/β type proteins and generally consists of a core domain and a subdomain^12^. The active site cavity is located between the two domains. As shown in Fig. 1A, L-DEX catalyses the hydrolytic dehalogenation through an enzyme-substrate (E-S) intermediate^13^. In contrast, the reported crystal structures of DL-DEXs, including DehI from *Pseudomonas putida* PP3^1^ and DL-DEX Mb from *Methylobacterium* sp. CPA1^14^, show that the enzyme is an α-helical hydrolase. The dehalogenation catalyzed by DL-DEX proceeds without an ester intermediate, while it is directly mediated by an activated water molecule (Fig. 1B)^15^. For D-DEX, a catalytic mechanism was inferred from sequence similarity to DL-DEX. It was investigated by MD simulations^1,16,17^, but a structure-guided study is unavailable.

D-DEXs have been used to produce L-2-chloropropionic acid as a chiral intermediate for the production of herbicides^18–20^. However, because of the low reactivity and stability, no commercial enzyme has been produced. Although the immobilization that covalently attaches HadD AJ1 which is a D-DEX from *Pseudomonas putida* strain AJ1/23 to the controlled-pore glass has been tested to improve its stability and increase its tolerance to a high substrate concentration, only a minor improvement was made^19^. Structural and functional information is necessary to rationally direct enzyme engineering to meet scientific and industrial needs. To unravel the molecular basis of D-DEX catalysis, the structure of substrate-free HadD AJ1 as well as the structure of the enzyme-product complex (E-P) were determined. The structure-guided elucidation of the catalytic mechanism of D-DEX is presented here.

## Results and Discussion

### Overall structure of HadD AJ1

**Figure 2:**
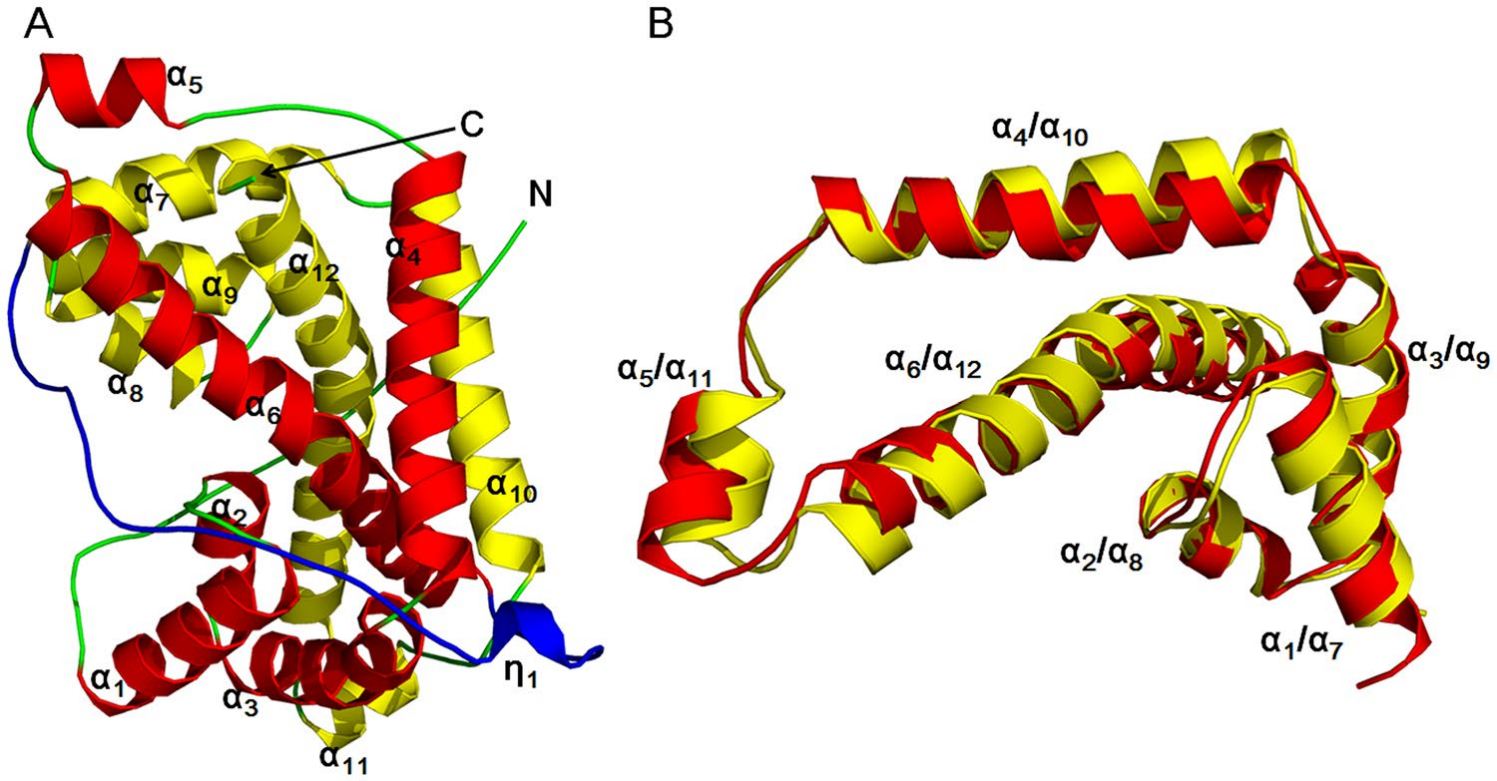
HadD AJ1 monomer. (**A**) Ribbon representation of monomeric HadD AJ1 composed of two repeats (repeat 1: red; repeat 2: yellow) and a linker (cyan). (**B**) 3D superposition of repeat 1 (red) and repeat 2 (yellow).

The crystal structure of the wild-type HadD AJ1 (WT) was determined by the molecular replacement method using the structure of a DL-DEX, DehI (PDB: 3BJX) from *Pseudomonas putida* PP3 as the search probe^1^. HadD AJ1 is refined at 2.64 Å resolution with an *R*_free_ of 25.1 (Table 1). HadD AJ1 is an α-helical hydrolase similar to DL-DEX. However, it completely differs from L-DEX, which is an α/β type hydrolase^1,12,14^. Each asymmetric unit of HadD AJ1 includes four monomers. Each monomer presents a compact fold featuring twelve α-helices and one 3_10_-helix η_1_ (Fig. 2B). Two structurally axisymmetric repeats are observed with 20% sequence identity and a superposition RMSD of 1.24 Å in each monomer. Repeat 1 and 2 consist of N-terminal α-helices 1–6 and C-terminal α-helices 7–12, respectively, linked by a lengthy loop with 33 residues including a 3_10_-helix η_1_ (Fig. 2). Each repeat is composed of a three-helix-bundle formed by the first three helices and a three-helix-triangular arrangement formed by the second three helices (Fig. 2B). The two repeats are organized by van der Waal forces, salt bridges, hydrogen bonds and hydrophobic interactions of α_6_ /α_12_ and α_4_ /α_10_, respectively. Helices α_4_ and α_10_ are spatially oriented parallel to each other. Helices α_6_ and α_12_ mutually interlace at their bulges, located in the midst of the helix. The similar symmetric architecture of the structurally repeated folds is also observed in the phosphorylation-coupled vitamin C transporter^21^. So far, the structurally internal repeats have been reported in many proteins, which are considered to be caused by genetic processes such as fusion and fission of domains, and gene duplication in protein evolution^22^.

**Table 1:** Statistics on data collection and refinement for X-ray analysis. ^a^WT: wild type HadD AJ1; ^b^E-P: the complex of WT with L-lactate.

Date set	WT^a^	E-P^b^
PDB ID	5H00	5GZY
Space group	*P*2_1_2_1_2_1_	*P*2_1_
Unit cell	a = 95.7 Å, b = 109.4 Å, c = 138.7 Å;	a = 72.9 Å, b = 95.1 Å, c = 109.1 Å;
	α = β = γ = 90°	α = γ = 90°, β = 98.1°
Wavelength (Å)	0.9777	0.9777
Resolution (Å)	45.24–2.64 (2.74–2.64)	47.57–2.18 (2.26–2.18)
Unique reflections	42453 (4042)	76730 (7281)
Multiplicity	8.4 (8.6)	6.7 (5.9)
Completeness (%)	97.8 (94.6)	99.4 (94.7)
Mean I/sigma (I)	9.73 (3.09)	9.78 (3.72)
R-merge (%)	14.3 (53.1)	13.9 (37.0)
R-meas (%)	15.2	15.1
Phasing and refinement		
Resolution (Å)	45.24–2.64 (2.70–2.64)	47.57–2.18 (2.21–2.18)
Number of reflections	1158	1168
*R*_work_/*R*_free_ (%)	19.3/25.1 (22.2/30.5)	15.8/19.7 (18.1/25.2)
Number of non-hydrogen atoms	9561	10832
Residues		
Macromolecules	9218	9304
ligands	0	24
Water	343	1504
RMSD bond (Å)	0.01	0.008
RMSD angle (°)	1.17	1.03
RMSD chiral (Å)	0.052	0.040
Ramachandran plot (%)		
Favored (%)	96.78	98.71
Allowed (%)	3.04	1.29
Outliers (%)	0.17	0
Average B-factor (Å^2^)	28.5	15
Macromolecules	28.5	13.6
Ligands	0	13.5
Solvent	28	23.8

**Figure 3:**
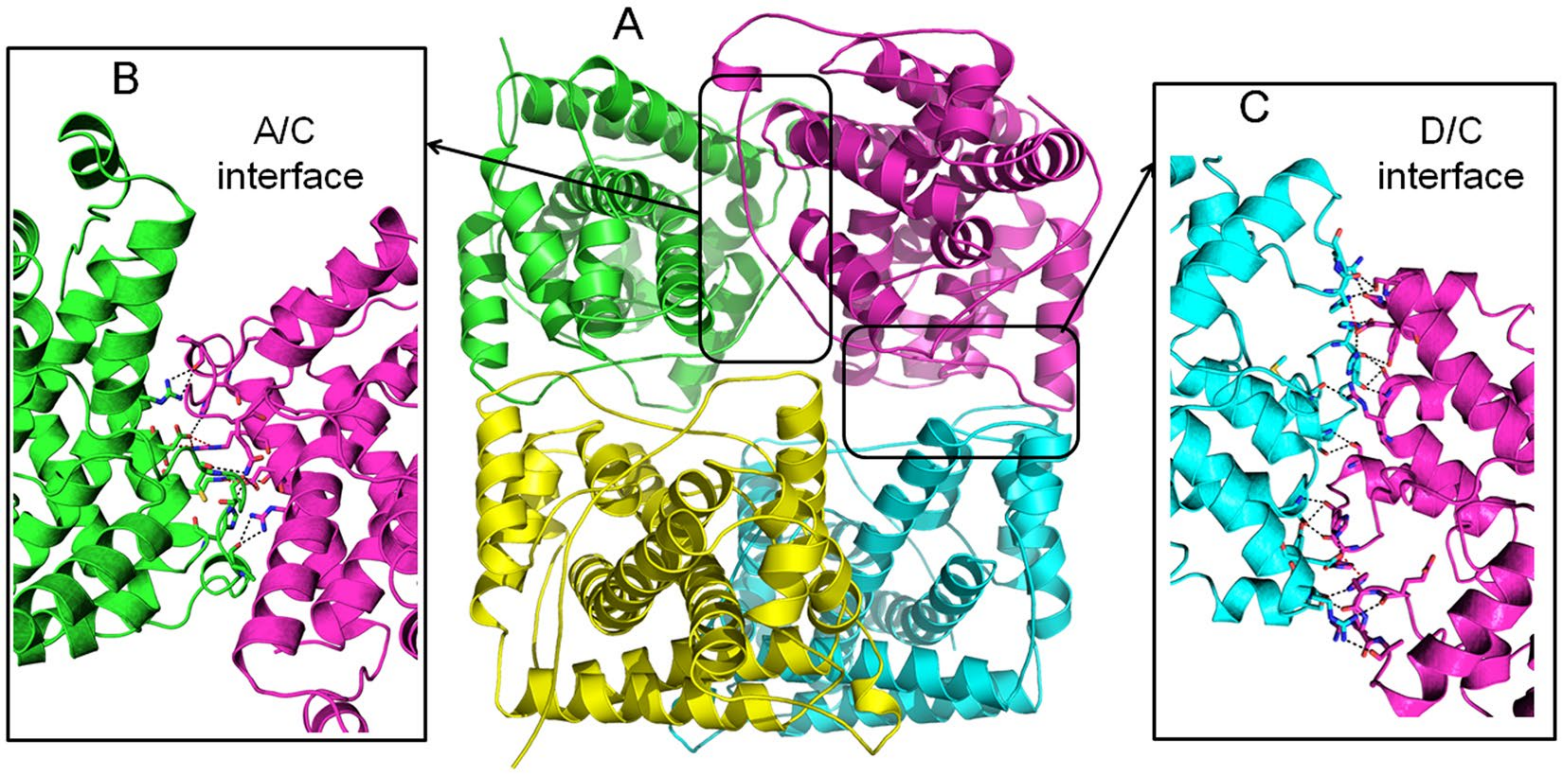
The whole structure of HadD AJ1. (**A**) Ribbon representation of tetrameric structure of HadD AJ1 including chain A (green), chain B (yellow), chain C (magenta) and chain D (cyan). Two interfaces are formed primarily through hydrogen bonds and salt bridge interactions (shown as short dashed lines). (**B**) A/C interface (**C**) D/C interface.

HadD AJ1 folds into a homotetramer in the crystal (Fig. 3), which is consistent with the functional state previously reported in solution^18^. In the tetramer, two types of interfaces including A/C (or B/D) and D/C (or A/B) are observed in the assembly of the monomers with the online interactive tool Protein Interfaces, Surfaces and Assemblies (PDBePISA, http://www.ebi.ac.uk/msd-srv/prot_int/cgi-bin/piserver). The interfaces are stabilized by hydrogen bonds and salt bridge interactions (Fig. 3B and C). Both interfaces comprise approximately 1000 Å^2^ (~12%) of total buried surface area, which is considered to be a weak association for oligomeric proteins^23,24^.

### Comparison of D-DEX and DL-DEX structures

**Figure 4:**
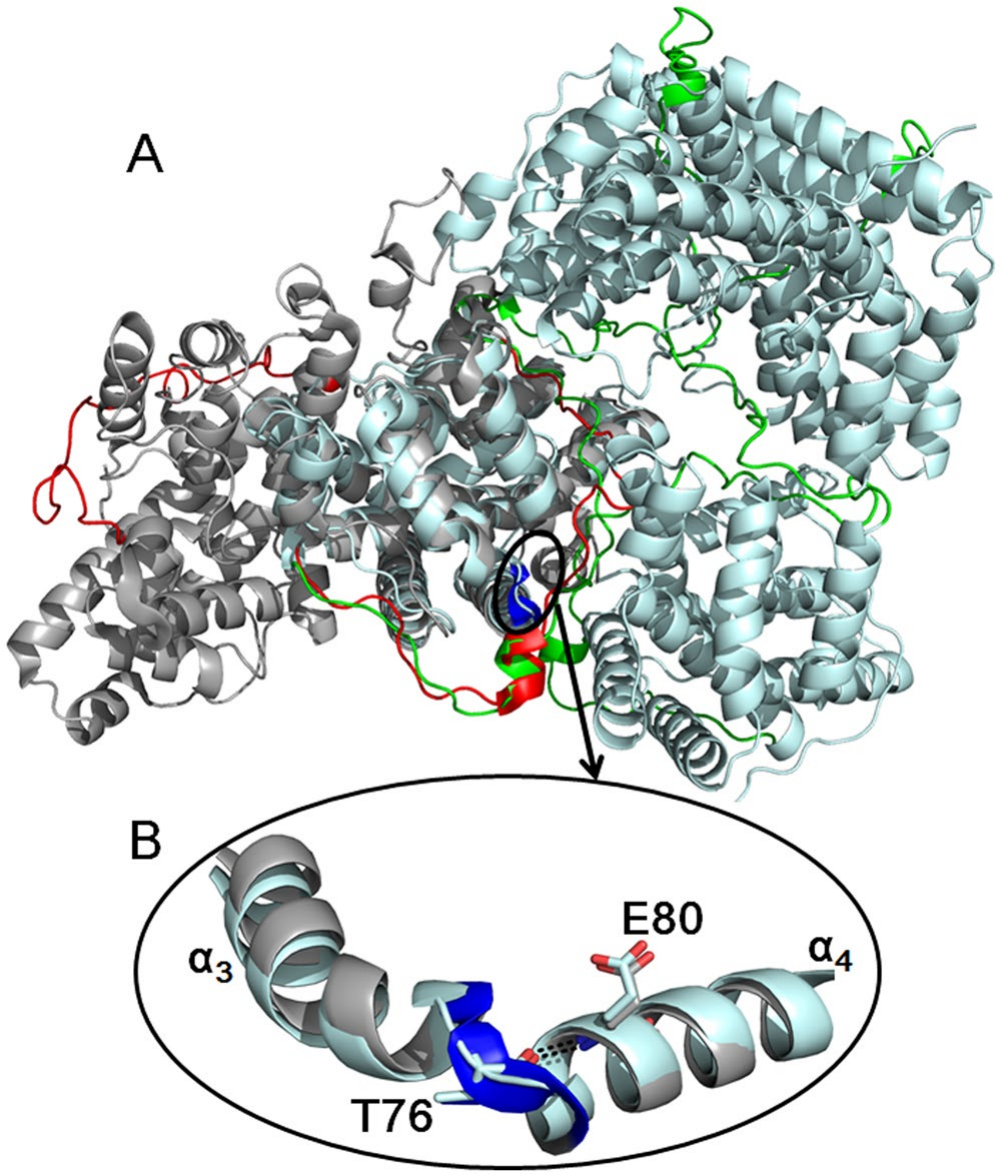
Comparison of HadD AJ1 and DehI structures. (**A**) Structural superposition of HadD AJ1 (pale cyan) and DehI (grey). (**B**) There is a different connection between α_3_ and α_4_ in both enzymes. The bend in DehI is highlighted by blue, and T76 and E80 are labelled in their sequentially corresponding positions in HadD AJ1. Hydrogen bond interactions are shown as short dashed lines.

D-DEXs share 30% similarity with DL-DEXs in primary structure (Supplementary Fig. S1). As shown in Fig. 4A, the superposition of HadD AJ1 with DehI reveals a very high degree of structural similarity with an RMSD of 1.05 Å for 207 C_α_ atoms^1^. The similar structural repeats are regarded as a “pseudo-dimer” in DehI. The “pseudo-dimers” of both enzymes overlap perfectly, also the linker. These similarities reveal the close evolutionary relationship between HadD AJ1 and DL-DEXs. However, two significant differences are observed. First, the η_1_ helix and the subsequent loop of the linker between the two repeats are buried in the interface assisting the assembly of the tetrameric HadD AJ1, while the linker wraps around the outside of the homodimeric DehI molecule, without involving the dimer interface. Second, helices α_3_ and α_4_ of HadD AJ1 are connected by a loop fragment; whereas, a helical bend links the α_3_ and α_4_ of DehI (Fig. 4B). The two joints contain a strictly conserved Thr between D-DEXs and DL-DEXs. The replacement of Thr by Ala completely damages DL-DEX activity^1,25^, from which Thr is considered as essential for the dehalogenation. However, the same mutant of HadD AJ1 still retains 87.8% activity (Supplementary Fig. S2A), which suggests that Thr76 is not crucial for the conformational stabilization and HadD AJ1 catalysis. The distinct effects on dehalogenation resulted from the same mutation of the conserved Thr indicates some differences in mechanisms of D-DEXs and DL-DEXs, which can be explained by the corresponding structural differences of the loop and the helical bend.

### D-DEX dehalogenation mechanism

**Figure 5:**
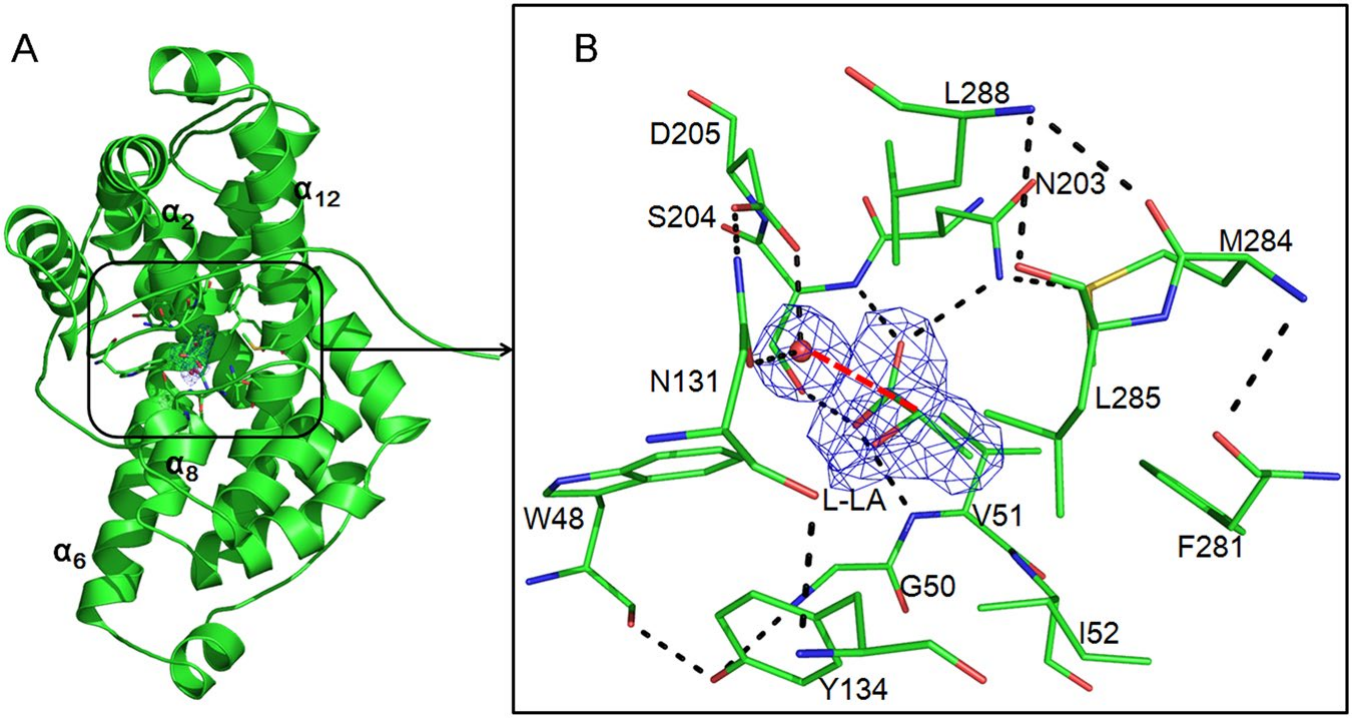
The structure of WT bound with L-LA. (**A**) Ribbon representation of HadD AJ1 including a water molecule and L-LA. Electron density (blue) corresponds to a 2*F*_*o*_*-F*_*c*_ map contoured at a level of 2σ. (**B**) Detailed view of L-LA and a catalytic water molecule staying in the active site in the E-P complex. Electron density (blue) corresponds to the 2*F*_*o*_*-F*_*c*_ map contoured at a level of 2σ. The water molecule is represented as a red ball. The red dashed line measures the distance between the catalytic water and the C2 atom of LA at 3 Å. Hydrogen bonds between the residues and L-LA are shown as black dashed lines.

To unveil the dehalogenation mechanism of D-DEX by exploring the molecular details of substrate recognition, HadD AJ1 was crystallized in the presence of the substrate D-2-chloropropionate (D-2-CPA) at pH5.5~6.4 and 277 K. In addition, co-crystallization and crystal soaking trials were undertaken with other reactive substrates including chloroacetate, bromoacetate, and 2-bromopropionate as well as with nonreactive substrates including 2-fluoropropionate, L-2-chloropropionate, 3-chloropropionate, 2,2-dichloropropionate and 2-bromo- 2-methylpropionate. Unfortunately, no E-S complex crystals were obtained. However, it turned out that HadD AJ1 was able to bind the product L-LA in the active site. The E-P complex was captured at 2.18 Å resolution (Fig. 5). Further inspection into the complex structure shows that L-LA is caged in the enclosed binding pocket of 71 Å^3^. The binding pocket is oriented at the interface of the two repeats and framed by the structurally repeat-related α_2_, α_8_, α_6_ and α_12_ (Fig. 4A). There are 13 amino acids arranged in the binding pocket which are Trp48, Lys50, Val51, Ile52, Asn131, Tyr134, Asn203, Ser204, Asp205, Phe281, Met284, Leu285 and Leu288 (Fig. 5B). The product-bound pocket has an identical spatial location to the active site of DehI (Supplementary Fig. S2B)^1^. By comparison of the active sites of HadD AJ1 and DehI, the residues corresponding to Trp48, Asn131, Tyr134, Ser204 and Asp205 are highly conserved between HadD AJ1 and DL-DEXs, whereas the remaining residues of the active sites are not (Supplementary Fig. S1), and their functions are discussed below.

Although another product of the catalyzed reaction - the departing Cl^−^ is missing in the complex, a water molecule adjacent to L-LA is trapped in the active site (Fig. 5B). This water molecule interacts with the carbonyl atom of the side chain of Asn131 and the carboxylic hydroxyl of Asp205 by forming hydrogen bonds. The distance between water and the C2 atom of L-LA is 3.0 Å. Considering the conservation of Asp205 with the catalytic residue Asp189 of DehI^1,25,26^, we propose that Asp205 and Asn131 are key residues that activate water required for the hydrolytic dehalogenation of HadD AJ1. Therefore, the mutation of Asp205 and Asn131 was performed (Fig. 5A). Notably, the replacement of Asp205 by Asn completely abolishes the enzymatic activity, which reveals that it’s a critical catalytic residue in HadD AJ1. Furthermore, residue Asp is strictly conserved among D-DEXs and DL-DEXs (Supplementary Fig. S1). The mutation of Asn131 to Asp in HadD AJ1 loses approximately 96% of catalytic activity, which shows Asn131 is an important residue for the dehalogenation. The location of the water molecule and its interactions with the two crucial residues Asp205 and Asn131 indicate that it is likely to be involved in the hydrolytic dehalogenation. Therefore, we hypothesize that the C2 atom of the substrate is directly attacked by a nucleophilic water molecule (Fig. 1B).

**Figure 6:**
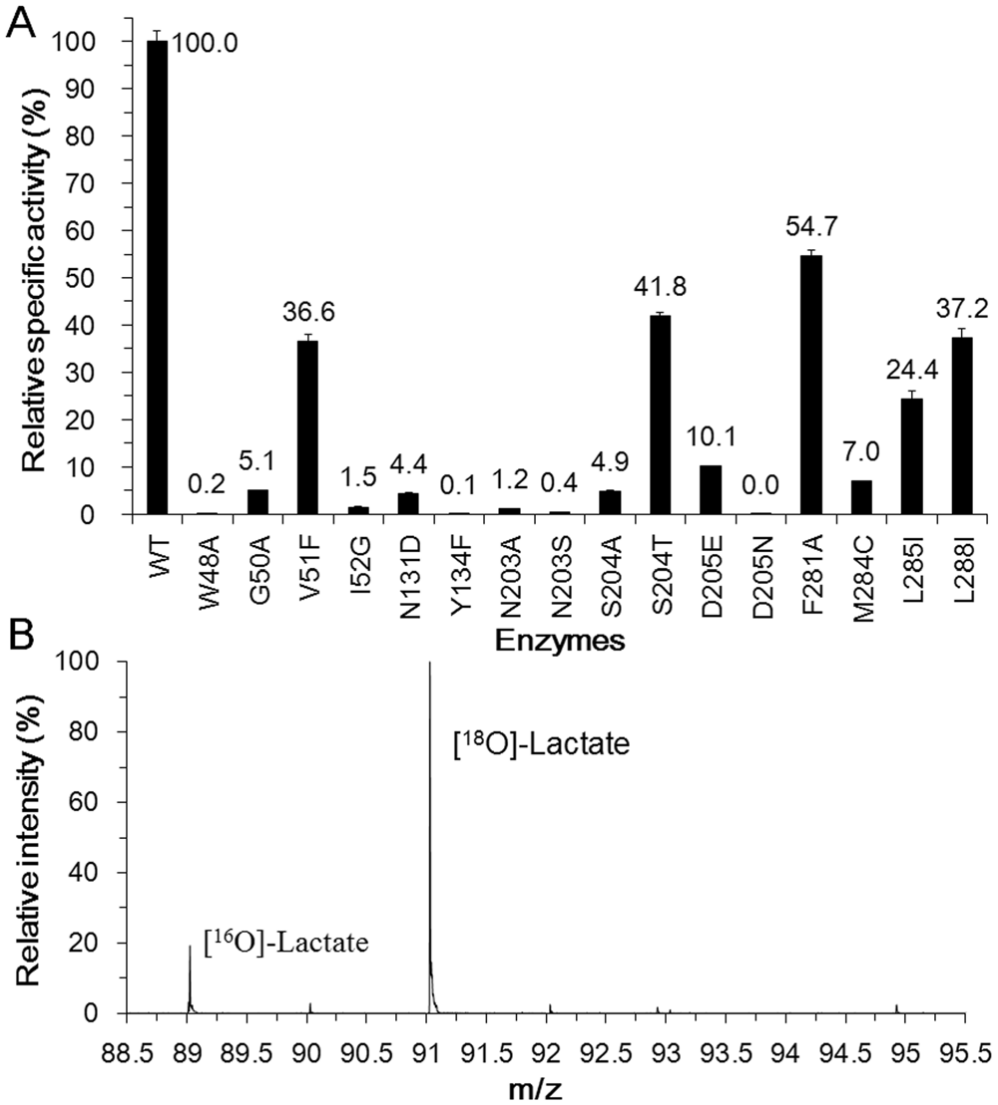
Enzymatic activity and mechanistic analyses of HadD AJ1. (**A**) Enzymatic activity analysis of HadD AJ1 and its mutants. Specific activity was determined with the purified enzyme as described in the methods and materials section. For WT enzyme and mutants V51F, S204T, F281Y, L285I and L288I, 0.02 mg of pure protein was used for the activity assay and 0.3 mg of pure protein was used for other mutants. The activity of the WT enzyme towards D-2-CPA was 31.9 U/mg protein. (**B**) Ion spray mass spectra of L-LA produced by a single turnover reaction of HadD AJ1 in H_2_^18^O.

To confirm our hypothesis, a single turnover reaction was conducted in H_2_^18^O with the WT enzyme that was ten times higher concentration than D-2-CPA. As seen in Fig. 6B, 5.2 times more labelled ^18^O-L-LA was produced compared to ^16^O-L-LA, which indicates that the dehalogenation is directly mediated by a water molecule. Furthermore, this finding confirms that the nucleophilic water molecule is activated by the Asp205 residue with the assistance of Asn131. The carbonyl oxygen in the side chain of Asp205 forms hydrogen bonds with the -NH_2_ group of Asn131, which drives Asp205 to attract a proton from a proximal water molecule. The activated water molecule is poised to attack the C2 atom of the substrate without involving an E-S ester intermediate, which is identical to DL-DEX but different from L-DEX^15^.

As stated above, the E-P complex structure is captured after releasing Cl^−^ and before L-LA departure from the active site. Hydrogen bonds and hydrophobic interactions predominantly stabilize the product in the active site. Hydrogen bonds are formed between the carboxyl of L-LA and the backbone amide nitrogen of Val51, the amino nitrogen of the side chain in Asn203, the backbone amide nitrogen of Ser204, and the carboxyl of Asp205 (Fig. 5B). Carboxyl is invariant in the substrate and the product before and after the dehalogenation. Therefore, it is hypothesized that Val51, Asn203, Ser204 and Asp205 contribute to stabilizing the substrate as well. Correspondingly, mutagenesis of these residues would be expected to affect enzymatic activity. As shown in Fig. 6A, 63.4%, 98.8%, 99.6%, 95.1% and 58.2% of enzymatic activity is lost for mutants V51F, N203A, N203S, S204A and S204T, respectively, towards D-2-CPA. The replacement of non-conserved Val51 and Asn203 by the equivalent residues found in DL-DEX led to a significantly reduced HadD AJ1 activity, which sheds light on different roles of these positions in the two types of enzymes. The increased *K*_m_ of the mutants V51F and N203A demonstrate their contribution to the binding interactions with the ground state substrates (Table 2). Consistent with the altered activity, N203A exhibits an obviously decreased *k*_cat_/*K*_m_ which is 355-fold lower than that of the WT enzyme (Table 2), which suggests the side chain of Asn203 is crucial for stabilizing the E-S transition state. By contrast, V51F exhibits a 4-fold lower *k*_cat_/*K*_m_ value than the WT enzyme (Table 2), which indicates that the increased steric hindrance in the mutant V51F does not severely perturb the transition state. The different loss in the enzymatic activity resulted from S204A and S204T reveals that the hydroxyl group of Ser204 and its orientation affects the interaction with the substrates. The D205E mutant retains 10.1% of activity (Fig. 6A), suggesting the carboxylic hydroxyl of Asp205 is essential for HadD AJ1 dehalogenation. Simultaneously, the increased *K*_m_ proves that Asp205 participates not only in water activation but also in the stabilization of the substrate (Table 2). Additionally, the thermodynamic contributions of Val51, Asn203 and Asp205 are demonstrated by the elevated transition state free energies of 3.5 kJ/mol, 14.8 kJ/mol and 6.49 kJ/mol resulting from V51F, N203A and D205E mutants, respectively, in comparison with that of WT (Table 2).

**Table 2:** Steady state kinetics of D-2-CPA dehalogenation by HadD AJ1 and its mutants. ^a^Difference in transition state free energies between WT and mutant enzymes. ΔΔG_ES_^*^ = −RTln[(*k*_cat_/*K*_m_)^WT^/(*k*_cat_/*K*_m_)^mutant^]^34^.

Enzymes	WT	V51F	N203A	D205E
*K*_m_ (mM)	0.94	2.90	12.00	4.13
*k*_cat_ (min^−1^)	981.00	757.00	35.40	330.00
(*k*_cat_/*K*_m_)_WT_/(*k*_cat_/*K*_m_)_mutant_	—	4.02	355.28	13.12
ΔΔG_ES_^*^(kJ/mol)^a^	—	−3.50	−14.80	−6.49

Residues Ile52, Phe281, Met284 and Leu285 form a hydrophobic pocket, which engages in hydrophobic interactions with the -CH_3_ group of L-LA. The mutations of Ile52 to Gly and Met284 to Cys resulted in 98.5% and 93% activity loss, respectively, suggesting the hydrophobic side chains of these two residues make a great contribution to the interaction with the substrate. The mutantations F281A and L285I destroy 45.7% and 75.6% of enzymatic activity, respectively, which indicates that hydrophobic residues of appropriate sizes are required in these positions for interacting with the substrate. The Cl^−^ is released towards Phe281 after the C2 atom is attacked from the opposite side of the halogen atom by the nucleophilic water molecule. Therefore, Phe281 was speculated to be involved in stabilizing the departing Cl^−^, which is likely to be trapped by an anion-π interaction between it and the phenyl group of Phe or a non-classical hydrogen bond, resulting from its “side-on” position at the plane of the benzene ring of Phe^27,28^. Consequently, the dehalogenation is affected by the mutation of Phe to Ala by destroying this interaction. The residues Trp48, Gly50, Tyr134 and Leu288 indirectly interact with L-LA, but they participate in the hydrogen-bond network among the residues in the active site. Residues Trp48 and Tyr134 are strictly conserved between HadD AJ1 and DL-DEXs. In agreement with the effects resulting from the mutation of the two residues in DL-DEXs^15^, HadD AJ1 mutants W48A and Y134F are almost inactive (Fig. 6A). Although Gly50 and Leu288 are replaced by the corresponding residues of DL-DEXs, the mutations damage 94.9% and 62.8% of HadD AJ1 activity, respectively (Fig. 6A). This result shows that the polarity and the steric hindrance of the residues at these positions have an impact on the dehalogenation catalyzed by HadD AJ1. These different contributions are likely to be caused by natural selection of the residues in the active site among D-DEXs and DL-DEXs.

## Conclusions

By combining structural, biochemical and site-directed mutagenesis analyses, we have gained insights into the dehalogenation catalyzed by D-DEX. HadD AJ1 is wholly α-helical and was crystallized as a homotetramer. Each monomer contains two N-terminal and C-terminal repeats, linked by a loop with a 3_10_-helix η1. The substrate binding pocket of HadD AJ1 is locatedat the interface between two repeats. Residues lining the active pocket participate in the E-S interaction, the mutation of which severely impairs the enzymatic activity. Dehalogenation catalyzed by D-DEX is directly mediated by a nucleophilic water molecule activated by Asp205 and Asn131 without forming the E-S ester intermediate, which is the same as DL-DEX but different from L-DEX. These findings enrich the knowledge on haloacid dehalogenases.

## Methods and Materials

### Cloning, expression and purification

The DNA sequence of HadD AJ1 was obtained from the European Nucleotide Archive (ENA), and the ENA code is AAA25831.1 (http://www.ebi.ac.uk/ena/data/view/AAA25831). The 909 bp *hadd aj1* gene was subcloned into a pET-28a vector for expression with a C-terminal hexa-His tag. This recombinant plasmid, with *Nco*I and *Xho*I as the restriction sites, was constructed by Invitrogen (Shanghai, China) and transformed into competent *Escherichia coli* DH5α. The recombinant protein was expressed in *E*. *coli* BL21 (DE3) cells and cultivated in Luria Broth media at 37 °C. Isopropyl-β-1-thiogalactopyranoside with a final concentration of 0.5 mM was added for another 4 hours when an optical density (at a wavelength of 600 nm) of 0.5 to 0.6 was reached. The overexpressed protein was then purified by nickel affinity chromatography (GE Healthcare) and gel-filtration chromatography using Superdex 200 (HiLoad 16/60, GE Healthcare). The purified WT enzyme was stored in Buffer A (50 mM Tris-HCl pH 8.0, 5% glycerol, 10 mM β-mercaptoethanol, and 1 mM EDTA). Additionally, all of the above enzymes were stored in Buffer B (25 mM KH_2_PO_4_-K_2_HPO_4_, pH 8.0). Protein concentration was determined using the Bradford assay, and the purity was analysed by sodium dodecyl sulfate polyacrylamide gel electrophoresis (SDS-PAGE).

### Crystal preparation

Crystals of HadD AJ1 WT and its mutants were obtained by the hanging-drop vapour diffusion method. For WT, a 3.3 μL drop, including 1.5 μL 4.3 mg/mL of protein, 1.5 μL of reservoir solution (RS I) including 0.1 M MES pH 6.43, 4% PEG20000, 10% PEG8000 and 0.3 μL of 1 M potassium sodium tartrate tetrahydrate (PST) was equilibrated against 500 μL of RS I. Crystals that grew approximately 16 days at 277 K were selected for diffraction. Crystals of WT with L-lactate (L-LA, the complex is designated as E-P) were prepared by co-crystallization using the hanging drop method. A 3.3 μL drop, including 1.5 μL of 6.2 mg/mL mM protein, 1.5 μL of RS I with 2 mM D-2-CPA, and 0.3 μL of 1 M PST, was equilibrated against 100 μL of RS I. Crystals were selected that had grown for approximately 27 days at 277 K. All selected crystals were preserved in liquid nitrogen before X-ray diffraction.

### X-ray diffraction analysis

X-ray diffraction data were collected on BL 18U1 and BL19U at Shanghai Synchrotron Radiation Facility (China). The diffraction data were processed and scaled using the HKL 2000 software package^29^. Then, the scaled output was converted into MTZ files using scalepack2mtz from the CCP4 suite^30^. The molecular replacement method was used to determine the structure of HadD AJ1 using Phaser-MR from the Phenix^31^ suite with DehI (PDB: 3BJX) as a search model^1^. Model rebuilding was performed in Coot^32^. Subsequently, further rounds of restrained refinement were performed, and water molecules were added and adjusted using the Phenix refine program. Structure figures were prepared with PyMol (http://www.pymol.org). Sequence comparison was generated by ESPript 3.0 ^33^. The structural information of all crystals is shown in Table 1.

### Site-directed mutagenesis of HadD AJ1

The residues involved in the substrate binding pocket were mutated. The residues Trp48 and Thr 76 which are strictly conserved between D-DEX and DL-DEX were mutated to Ala. The residues Gly50, Val51, Ile52, Asn203, Phe281, Leu285 and Leu288 which are not conserved between D-DEX and DL-DEX were mutated to the corresponding amino acids found in DehI PP3. The residues Asn131, Tyr134, Ser204, Asp205 and Met284 which are identical between HadD AJ1 and DL-DEX were mutated on the basis of their charge and polarity. The primers containing mutations were designed using DNAstar and are summarized in Supplementary Table S2. HadD AJ1 was mutated with the Prime Star Mix (TaKaRa Bio, Dalian, China) according to the manufacturer’s instructions and verified by sequencing. The plasmids were transferred into *E*. *coli* BL21 (DE3). The expression and purification conditions were the same as that of the wild type, unless stated otherwise. The SDS-PAGE analysis of the purified protein was shown in Supplementary Fig. S3

### Single turnover reaction of HadD AJ1 in H_2_^18^O

First, 200 nmol of HadD AJ1 in 25 mM Tris-HCl, pH 8.0, were lyophilized. The reaction was initiated by dissolving the dried enzyme in 300 μL H_2_^18^O containing 39 mM Glycine-NaOH, pH 10.0, and 20 nmol D-2-CPA. The reaction mixture was ultrafiltered after the incubation at 30 °C for 18 h and was then directly injected into the mass spectrometer. The molecular mass of the produced lactate was measured in the quadrupole time-of-flight mass spectrometry (Agilent Technologies 6540 UHD Accurate-Mass Q-TOF, USA) equipped with an electrospray ionization ion source in the negative mode.

### Enzymatic activity

The enzymes in Buffer D were used to assay the activity towards D-2-CPA. The standard assay system was used unless stated otherwise^6^. The reaction mixture (1 mL) contained 10 mM D-2-CPA, 100 mM glycine-NaOH (pH 10.0), and enzyme. To ensure that at least 5% of the substrate is degraded, the enzyme concentrations were 0.02 mg/mL for WT, V51F, S204T, F281Y, L285I and L288I, and 0.3 mg/mL for other mutants. The reactions were terminated by the addition of 10 µL of phosphoric acid (85% w/w) following the incubation at 30 °C. After the precipitates were removed by centrifugation (14,000 × g, 10 min), the supernatants were analyzed by HPLC to determine the D-2-CPA contents. One unit of dehalogenase activity was defined as the amount of enzyme that catalyzed the hydrolysis of 1 µmol D-2-CPA per min.

### Steady-state kinetic measurements

Kinetic study of WT and several mutants was carried out by assaying the activity of the enzyme under standard condition at varying initial substrate concentrations. The initial rates of the reaction at different substrate concentrations were measured. Kinetic equation fitting was conducted using Origin 7 software. *K*_m_ and *k*_cat_ values were derived from the non-linear regression analysis (Supplementary Fig. S4).

### Data Availability

All data generated or analysed during this study are included in this published article (and its Supplementary Information files).

## Electronic supplementary material

Supplementary Information

## References

[1] Schmidberger JW, Wilce JA, Weightman AJ, Whisstock JC, Wilce MCJ (2008). The Crystal structure of DehI reveals a new α-haloacid dehalogenase fold and active-site mechanism. J. Mol. Biol..

[2] Swanson PE (1999). Dehalogenases applied to industrial-scale biocatalysis. Curr. Opin. Biotechnol..

[3] Furukawa K (2006). Oxygenases and dehalogenases: Molecular approaches to efficient degradation of chlorinated environmental pollutants. Biosci. Biotechnol. Bioch..

[4] Huyop F, Sudi IY (2012). D-specific dehalogenases, a review. Biotechnol. Biotechnol. Eq..

[5] Kasai N, Suzuki T, Furukawa Y (1998). Chiral C3 epoxides and halohydrins: Their preparation and synthetic application. J. Mol. Catal. B: Enzym..

[6] Zhang J, Xin Y, Cao X, Xue S, Zhang W (2014). Purification and characterization of 2-haloacid dehalogenase from marine bacterium *Paracoccus* sp. DEH99, isolated from marine sponge *Hymeniacidon perlevis*. J. Ocean U. China.

[7] Marzorati M (2010). Bacterial diversity and reductive dehalogenase redundancy in a 1,2-dichloroethane-degrading bacterial consortium enriched from a contaminated aquifer. Microb. Cell Fact..

[8] Janssen DB, Pries F, Van Der Ploeg JR (1994). *Genetic and biochemistry of dehalogenating enzymes*. Annu. Rev. Microbiol..

[9] Fetzner S (1998). Bacterial dehalogenation. Appl. Microbiol. Biotechnol..

[10] Rossberg, M. *et al*. Chlorinated Hydrocarbons in Ullmann’s Encyclopedia of Industrial Chemistry. *Wiley-VCH Verlag GmbH & Co*. *KGaA* 1–186 (2000).

[11] Hill KE, Marchesi JR, Weightman AJ (1999). Investigation of two evolutionarily unrelated halocarboxylic acid dehalogenase gene families. J. Bacteriol..

[12] Hisano T (1996). Crystal structure of L-2-haloacid dehalogenase from *Pseudomonas* sp YL: An alpha/beta hydrolase structure that is different from the alpha/beta hydrolase fold. J. Biol. Chem..

[13] Liu JQ, Kurihara T, Miyagi M, Esaki N, Soda K (1995). Reaction-mechanism of L-2-haloacid dehalogenase of *Pseudomonas* sp. YL: Identification of Asp(10) as the active-site nucleophile by O-18 incorporation experiments. J. Biol. Chem..

[14] Omi R (2007). Expression, purification and preliminary X-ray characterization of DL-2-haloacid dehalogenase from *Methylobacterium* sp. CPA1. Acta Crystallogr. Sect. F: Struct. Biol. Commun..

[15] Nardi-Dei V (1999). DL-2-haloacid dehalogenase from *Pseudomonas* sp. 113 is a new class of dehalogenase catalyzing hydrolytic dehalogenation not involving enzyme-substrate ester intermediate. J. Biol. Chem..

[16] Leigh JA, Skinner AJ, Cooper RA (1988). Partial purification, stereospecificity and stoichiometry of three dehalogenases from a *Rhizobium* species. FEMS Microbiol. Lett..

[17] Sudi IY (2012). Structure Prediction, Molecular Dynamics Simulation and Docking Studies of D-Specific Dehalogenase from *Rhizobium* sp. RC1. Int. J. Mol. Sci..

[18] Smith JM, Harrison K, Colby J (1990). Purification and characterization of D-2-haloacid dehalogenase from *Pseudomonas putida* strain AJ1/23. J. Gen. Microbiol..

[19] Parker K, Colby J (1995). Immobilization of the D-2-haloacid dehalogenase from *Pseudomonas putida* strain AJ1/23. Biodegradation.

[20] Taylor, S. C. D-2-haloalkanoic acid halidohydrolase. US 4758518 (1988).

[21] Luo P (2015). Crystal structure of a phosphorylation-coupled vitamin C transporter. Nat. Struct. Mol. Biol..

[22] Blaber M, Lee J, Longo LM (2012). Emergence of symmetric protein architecture from a simple peptide motif: evolutionary models. Cell. Mol. Life Sci..

[23] Ali MH, Imperiali B (2005). Protein oligomerization: How and why. Bioorg. Med. Chem..

[24] Nooren IMA, Thornton JM (2003). Structural characterisation and functional significance of transient protein–protein interactions. J. Mol. Biol..

[25] NardiDei V, Kurihara T, Park C, Esaki N, Soda K (1997). Bacterial DL-2-haloacid dehalogenase from *Pseudomonas* sp. strain 113: Gene cloning and structural comparison with D- and L-2-haloacid dehalogenases. J. Bacteriol..

[26] Siwek A (2013). Binding modes of DL-2-haloacid dehalogenase revealed by crystallography, modeling and isotope effects studies. Arch. Biochem. Biophys..

[27] Guo C (2015). Exploring the enantioselective mechanism of halohydrin dehalogenase from *Agrobacterium Radiobacter* AD1 by iterative saturation mutagenesis. Appl. Environ. Microbiol..

[28] Giese M, Albrecht M, Rissanen K (2016). Experimental investigation of anion–π interactions – applications and biochemical relevance. Chem. Comm..

[29] Otwinowski Z, Minor W (1997). Processing of X-ray diffraction data collected in oscillation mode in Methods in Enzymology. Academic Press.

[30] Winn MD (2011). Overview of the CCP4 suite and current developments. Acta Crystallogr. Sect. D: Biol. Crystallogr..

[31] Adams PD (2010). PHENIX: a comprehensive Python-based system for macromolecular structure solution. Acta Crystallogr. Sect. D: Biol. Crystallogr..

[32] Emsley P, Cowtan K (2004). Coot: model-building tools for molecular graphics. Acta Crystallogr. Sect. D: Biol. Crystallogr..

[33] Robert X, Gouet P (2014). Deciphering key features in protein structures with the new ENDscript server. Nucleic Acids Res..

[34] Copeland, R. A. Kinetics of Single-Substrate Enzyme Reactions in Enzymes. John Wiley & Sons, Inc. 109–145 (2002).

