# Supplementary material for: Insights into the molecular mechanism of dehalogenation catalyzed by D-2-haloacid dehalogenase from crystal structures
Source: Sci Rep. 2018 Jan 23;8:1454. doi: 10.1038/s41598-017-19050-x (PMC5780510; doi:10.1038/s41598-017-19050-x)
Supplement: Supplementary file 1 — Supplementary Information [file 41598_2017_19050_MOESM1_ESM.pdf]

Supporting Information

Insights into the molecular mechanism of dehalogenation catalyzed by D-2-haloacid dehalogenase from crystal structures

Yayue Wang,[1,2] Yanbin Feng,[1] Xupeng Cao,[1] Yinghui Liu,[1] Song Xue,[*1]

[1]Marine Bioengineering Group, Dalian Institute of Chemical Physics, Chinese Academy of Sciences, Dalian 116023, China. E-mail: xuesong@dicp.ac.cn

[2]University of Chinese Academy of Sciences, Beijing 100049, China

Contents

Supplementary figures

Supplementary Fig. S1. Sequence alignment of D-DEXs with DL-DEXs.

Supplementary Fig. S2. Structural superimposition of active sites between HadD AJ1 (cyan) and DehI (magenta, PDB: 3BJX).

Supplementary Fig. S3. SDS-PAGE of the purified proteins.

Supplementary Fig. S4. Plots of reaction velocities of WT and mutant enzymes.

Supplementary tables

Table S1. Mutant enzymes, base changes in the relevant triplets, and synthetic mutagenic primers used.

Supplementary figures

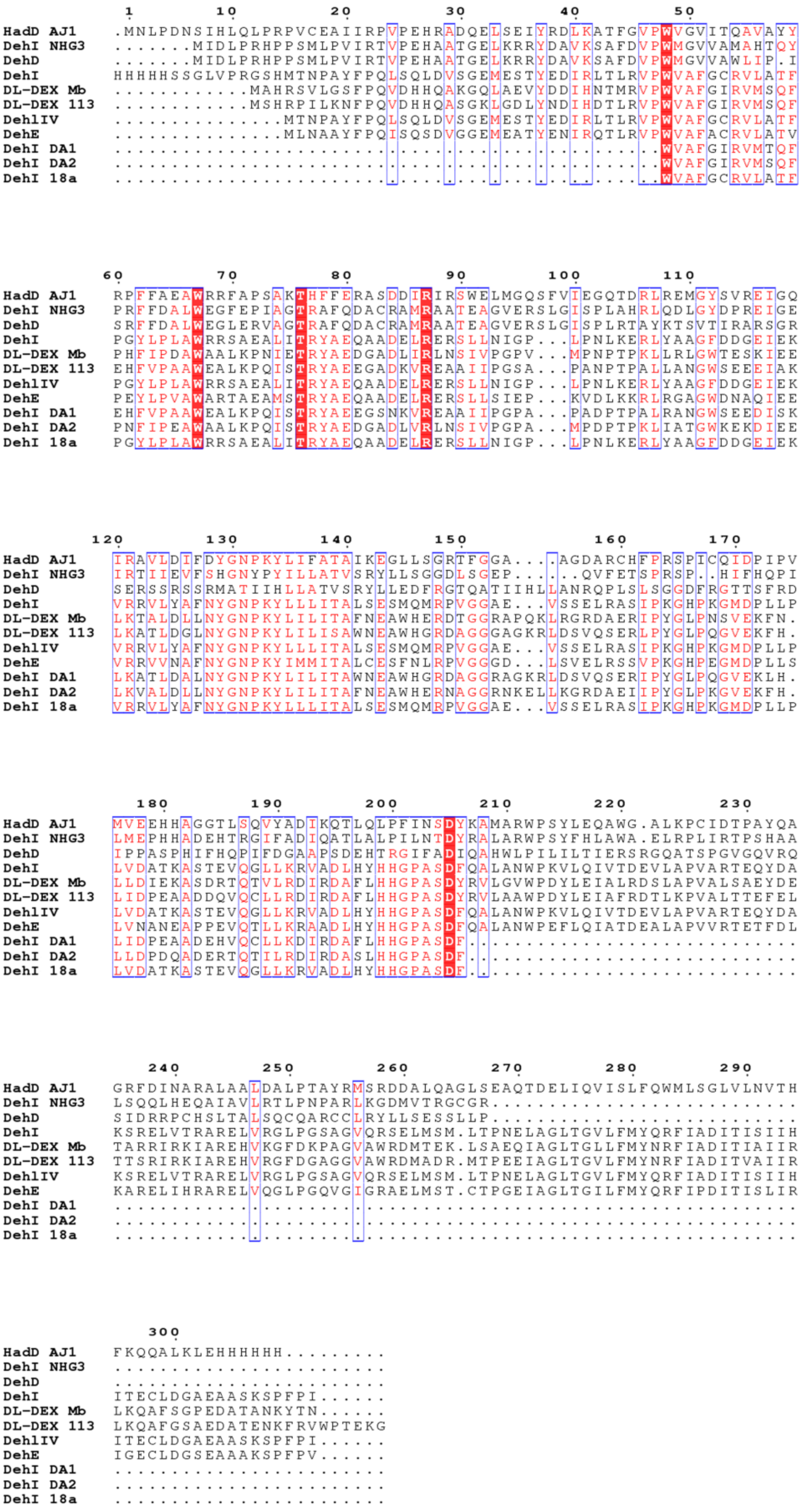

Supplementary Fig. S1. Sequence alignment of D-DEXs with DL-DEXs. D-DEXs include HadD AJ1 (UniProtKB entry: Q52086) from *Pseudomonas putida* AJ1/23, DehI NHG3 (GenBank: CAD32752.1) from *Agrobacterium* sp. strain NHG3 and DehD (GenBank: CAA63793.1) from *Rhizobium* sp. RC1. The amino acid sequences of DL-2-haloacid dehalogenases (DL-DEXs) are obtained from UniProt database (http://www.uniprot.org/) and EMBL database (http://www.ebi.ac.uk/ena). DL-DEXs include DehI (UniProtKB entry: Q8GJ84) from *Pseudomonas putida* pp3, DL-DEX Mb (UniProtKB entry: A6BM74) from *Methylobacterium* sp. CPA1, DL-DEX 113 (UniProtKB entry: O06652) from *Pseudomonas* sp. 113, DehIIV (UniProtKB entry: Q59168) from *Alcaligenes xylosoxydans* subsp. *xylosoxydans* ABIV, DehE (UniProtKB entry: Q52087) from *Pseudomonas putida* AJ1, DehI DA1 (EMBL accession: AJ133455) from *Bradyrhizobium* sp. strain DA1, DehI DA2 (EMBL accession: AJ133456) from *Bradyrhizobium* sp. strain DA2 and DehI 18a (EMBL accession: AJ133458) from *Bradyrhizobium* sp. strain 18a. Strictly conserved residues are highlighted red with white characters. The Clustal Omega[1] program is used for multiple sequence alignment (http://www.ebi.ac.uk/Tools/msa/clustalo/) and sequence similarities are analysed by ESPript program (http://espript.ibcp.fr/ESPript/ESPript/).[2] Identical and similar residues are shown in red characters.

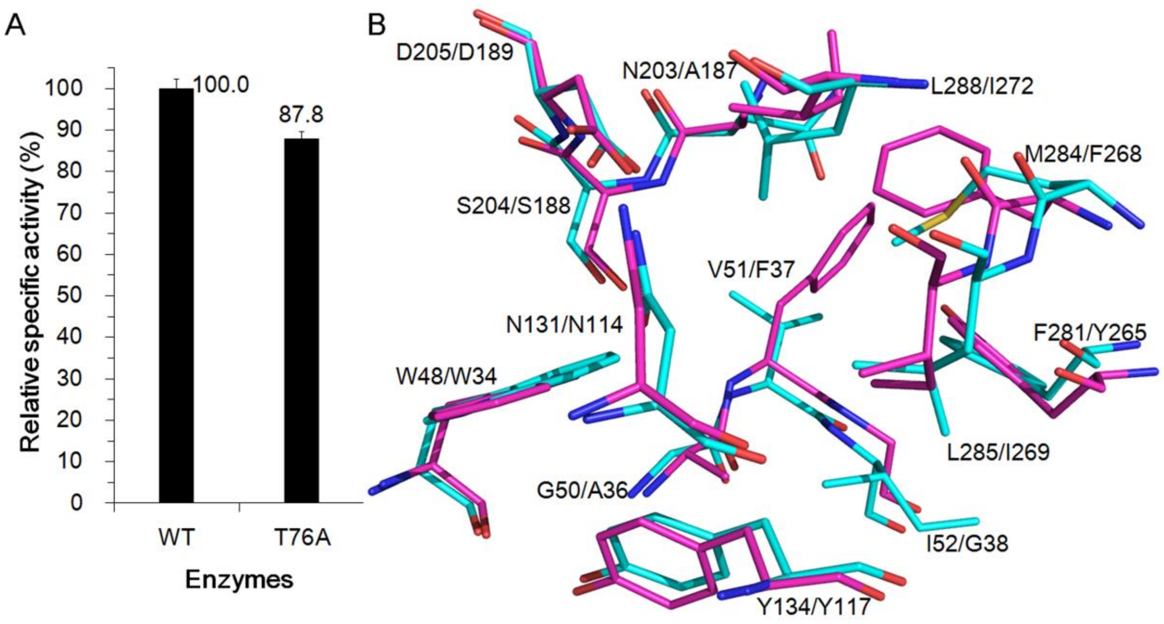

Supplementary Fig. S2. Enzymatic activity analysis and structural comparison. (A) Enzymatic activity analysis of HadD AJ1 and T76A mutant**.** (B) Structural superimposition of active sites between HadD AJ1 (cyan) and DehI (magenta, PDB: 3BJX). The former and the later in the amino acids presentation are the corresponding residues of HadD AJ1 and DehI, respectively. For example, V51/F37, V51 and F37 are the residues of HadD AJ1 and DehI, respectively.

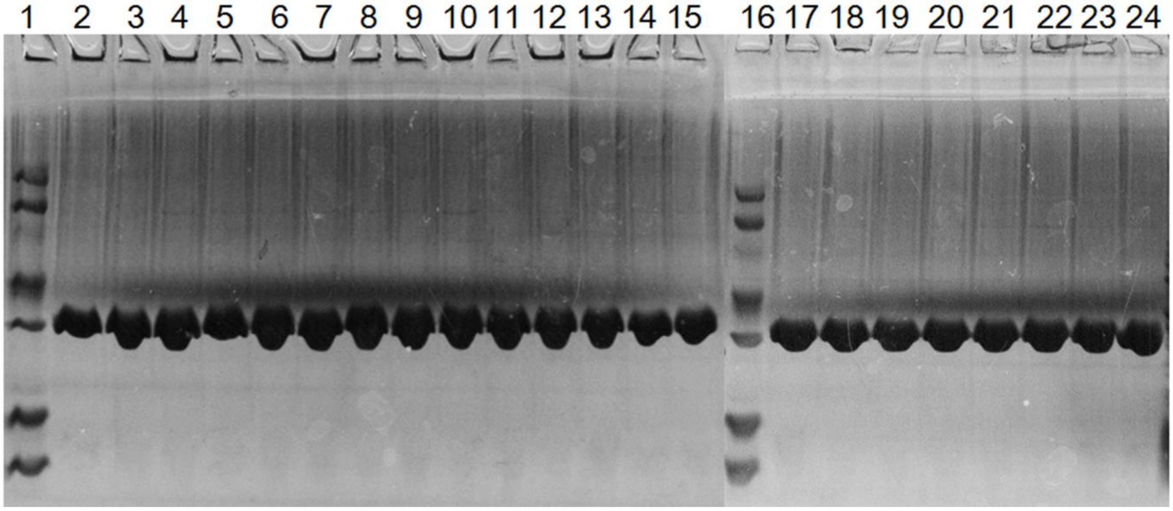

Supplementary Fig. S3 SDS-PAGE of the purified protein. 1 and 16, Marker, from top to bottom, the molecular weights are 97.2 kDa, 66.4 kDa, 44.3 kDa, 29.3 kDa, 20.1 kDa and 14.3 kDa in order; 2, WT; 5, G50A mutant; 6, V51F mutant; 7, I52G mutant; 8, T76A; 10, N131D mutant; 11, Y134F mutant; 12, N203A mutant; 13, N203S mutant; 15, S204T mutant; 17, WT; 18, D205E mutant; 19, D205N mutant; 21, F281A mutant; 22, M284C mutant; 23, L285I mutant; 24, L288I mutant. Lane 3, 4, 8, 9, 14 and 20 are other proteins.

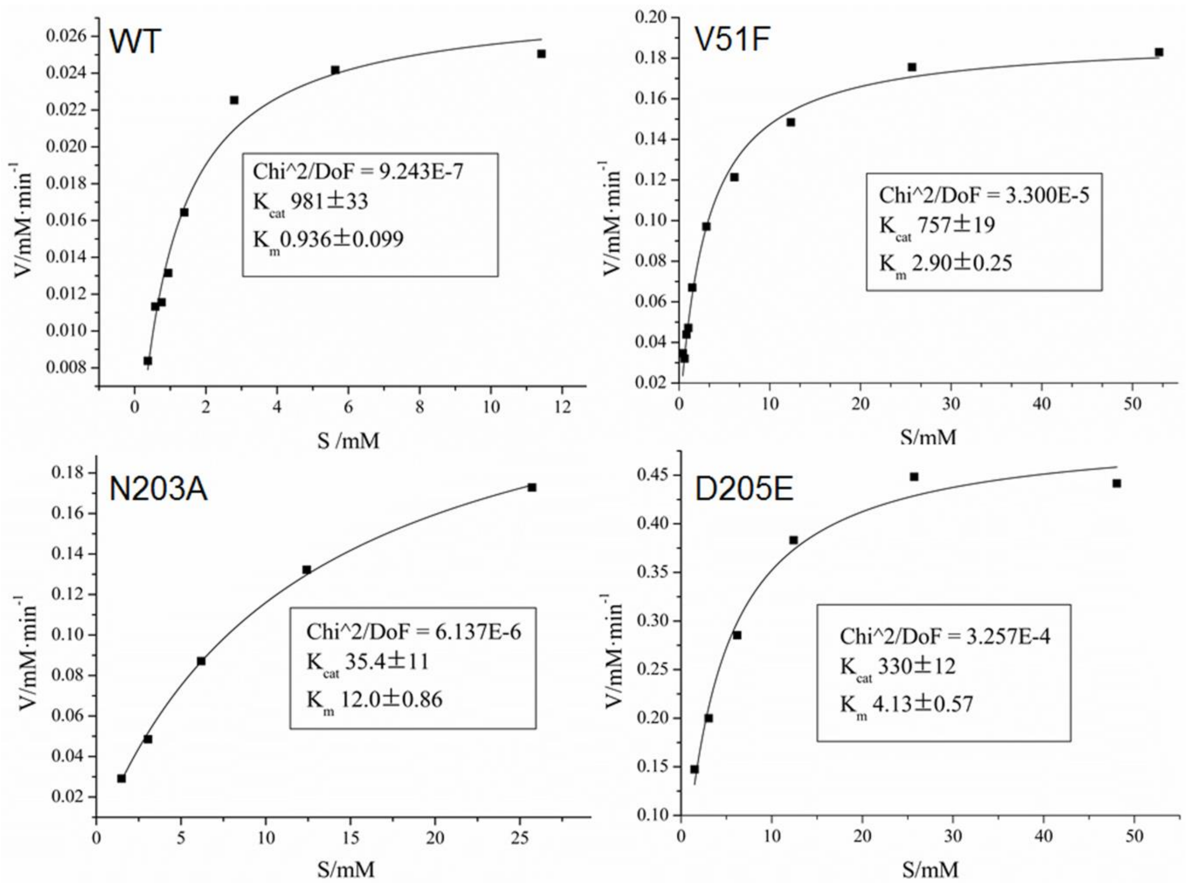

Supplementary Fig. S4. Plots of reaction velocities of WT and mutant enzymes

Supplementary tables

**Mutant enzyme**	**Mutation**	**Primer[a]**
W48A	TGG→GCG	5'-CGTTCGGCGTGCCCGCGGTTGGGGTCATCAC-3'
G50A	GGG→GCG	5'-CGTGCCCTGGGTTGCGGTCATCACGCAGGCG-3'
V51F	GTC→TTC	5'-GTGCCCTGGGTTGGGTTCATCACGCAGGCGG-3'
I52G	ATC→GGC	5'-CCCTGGGTTGGGGTCGGCACGCAGGCGGTCG-3'
T76A	ACC→GCC	5'- GCACCGTCTGCGAAAGCCCATTTTTTCGAGC-3'
N131D	AAC→GAC	5'-ATCTTCGATTACGGCGACCCGAAATATCTGA-3'
Y134F	TAT→TTT	5'-ACGCCAACCCGAAATTTCTGATTTTCGCCAC-3'
N203A	AAC→GCC	5'-AACTGCCGTTCATCGCCTCTGACTACAAGGC-3'
N203S	AAC→TCC	5'-AACTGCCGTTCATCTCCTCTGACTACAAGGC-3'
S204A	TCT→GCT	5'-CTGCCGTTCATCAACGCTGACTACAAGGCGA-3'
S204T	TCT→ACT	5'-CTGCCGTTCATCAACACTGACTACAAGGCGA-3'
D205E	GAC→GAG	5'-GTTCATCAACTCTGAGTACAAGGCGATGGCG-3'
D205N	GAC→AAC	5'-CCGTTCATCAACTCTAACTACAAGGCGATGG-3'
F281A	TTC-TGG	5'- AGGTTATCAGCCTGTGGCAATGGATGCTGTC-3'
M284C	ATG→TGC	5'-GCCTGTTCCAATGGTGCCTGTCTGGTCTGGT-3'
L285I	CTG→ATC	5'-TGTTCCAATGGATGATCTCTGGTCTGGTTCT-3'
L288I	CTG→ATC	5'-GGATGCTGTCTGGTATCGTTCTGAACGTTAC-3'

Table S1. Mutant enzymes, base changes in the relevant triplets, and synthetic mutagenic primers used. [a]Mutagenized nucleotides are underlined.

References

1. Li, W. et al. The EMBL-EBI bioinformatics web and programmatic tools framework. *Nucleic Acids Res.* 2015, **43**, 580-584 (2015).
2. Robert, X. & Gouet, P. Deciphering key features in protein structures with the new ENDscript server. *Nucleic Acids Res.* **42**, 320-324 (2014).
